# Advances in single-cell sequencing technology in microbiome research

**DOI:** 10.1016/j.gendis.2023.101129

**Published:** 2023-09-28

**Authors:** Yinhang Wu, Jing Zhuang, Yifei Song, Xinyi Gao, Jian Chu, Shuwen Han

**Affiliations:** a Huzhou Central Hospital, Affiliated Central Hospital Huzhou University, Huzhou, Zhejiang 313000, China; b Key Laboratory of Multiomics Research and Clinical Transformation of Digestive Cancer of Huzhou, Huzhou, Zhejiang 313000, China; c The Fifth Affiliated Clinical Medical College of Zhejiang Chinese Medical University, Hangzhou, Zhejiang 313000, China; d Zhejiang Provincial People's Hospital and Affiliated People's Hospital, Hangzhou Medical College, Hangzhou, Zhejiang 310000, China

**Keywords:** Bacterial antibiotic resistance, Host immunity, Host-phage interaction, Microbial single-cell sequencing, Microorganisms, Single cell, Single-cell sequencing technology

## Abstract

With the rapid development of histological techniques and the widespread application of single-cell sequencing in eukaryotes, researchers desire to explore individual microbial genotypes and functional expression, which deepens our understanding of microorganisms. In this review, the history of the development of microbial detection technologies was revealed and the difficulties in the application of single-cell sequencing in microorganisms were dissected as well. Moreover, the characteristics of the currently emerging microbial single-cell sequencing (Microbe-seq) technology were summarized, and the prospects of the application of Microbe-seq in microorganisms were distilled based on the current development status. Despite its mature development, the Microbe-seq technology was still in the optimization stage. A retrospective study was conducted, aiming to promote the widespread application of single-cell sequencing in microorganisms and facilitate further improvement in the technology.

## Introduction

Microorganisms are ubiquitous in human life. Human microbes play an important regulatory role in physiological development and human health. For example, gut microbes provide a key function in the fermentation of indigestible substances such as dietary fiber^1^ and endogenous intestinal mucus.^2^ Specific microorganisms that produce short-chain fatty acids and gases are combined with fermentation to produce butyric acid that could supply energy to human colon cells,^3^ propionic acid that is involved in regulating gluconeogenesis and satiety signals,^3^ and acetic acid that acts on cholesterol metabolism and lipogenesis.^4^ Additionally, factors produced by the microbiome strengthen intraepithelial tight junctions, increase mucus, stimulate wound repair, and promote stem cell proliferation. For instance, skin symbiotic microbiota could regulate skin and repair barrier function by regulating epithelial barrier genes (cytokeratin-10, tight junction protein 3, desmogleins 1a-b, and claudin-1) and affecting the signal transduction of epithelial aromatics receptors.^5^ The intestinal symbiotic microbiome that produces a variety of metabolites, including SCFA, secondary bile acids, and bacteriocins plays a role in resisting colonization by exogenous microorganisms.^6^ Research has revealed that many diseases arise from the loss and imbalance in healthy host–microbe interactions in the human body. In immune regulation, specific microorganisms can produce metabolites or express molecules that direct inflammatory or tolerogenic immune pathways, and function in the skin^7^, gut,^6^ lungs,^8^ and mouth^9^ where microorganisms reside. Microbial application was used in various fields of research, including medicine, food safety,^10^^,^^11^ and biogenetics,^12^ and in combination with bioinformatics^13^ and biochemistry tools for targeted therapies.^14^^,^^15^

Microorganisms can usually be detected as microbiomes, such as metagenomics, metatranscriptomes, and 16SeRNA. The microbiome is the sum of the genomes of microorganisms, including bacteria, archaea, fungi, and viruses, as well as their surrounding environment.^16^ Recently, some techniques that can detect single cells of microorganisms, such as microbial single-cell sequencing (Microbe-seq) and microbial split-pool ligation transcriptomics, have emerged. The microbiome is the genomic collection of all microorganisms and their genetic information in a given environment or ecosystem, whereas the microbial single cell is the individual genotype and functional expression of a microorganism, which is different from the population concept of the microbiome.

Transcriptome sequencing was pioneered in microbiomics research, aiming to the sum of all the RNAs, including mRNAs and non-coding RNAs, and a given cell can transcribe in a given functional state. Later, to acquire its structure and function, whole-genome sequencing was introduced to obtain the whole genome sequence of microbes by sequencing and assembling the genome.^17^ In addition to taxonomic information and more genetic information and functional genes enriched in specific environments, whole genome sequencing contains all the genetic information of a microorganism. However, microbes can heterogeneously activate gene expression programs in response to environmental changes, stress, and other stimuli. Even isogenic microorganisms often exhibit functional subsets that are essential for fitness and survival. The lack of single-cell resolution and the inability to identification of single-cell function were the limitations of microbiome sequencing technology.^18^ Different from whole-genome sequencing, Microbe-seq is a technique that detects the gene sequence of a microorganism at the single-cell level, which realizes the annotation of the microbial genome and the functional study of individual microbial genes. In the field of microbiology, single-cell sequencing (SCS) is currently being used in the areas of bacterial drug resistance, biological evolution, and elucidation of microbial dark matter.^19^, ^20^, ^21^

The history of microbial detection technology, the principles of Microbe-seq, the characteristics of Microbe-seq, and the application prospects for Microbe-seq were reviewed. Given the increasing importance of heterogeneity in microbial research, the authors hope to apply SCS to a wide range of microorganisms. SCS in microbes is promised to function in clinical diagnosis and clinical medication and contribute to exploring the mechanism of microorganisms affecting human health from the strain level. Besides, the technology is still immature and the authors expect more research to pay more attention to it and make efforts to improve it.

## The development process of microbial detection technology

**Figure 1:**
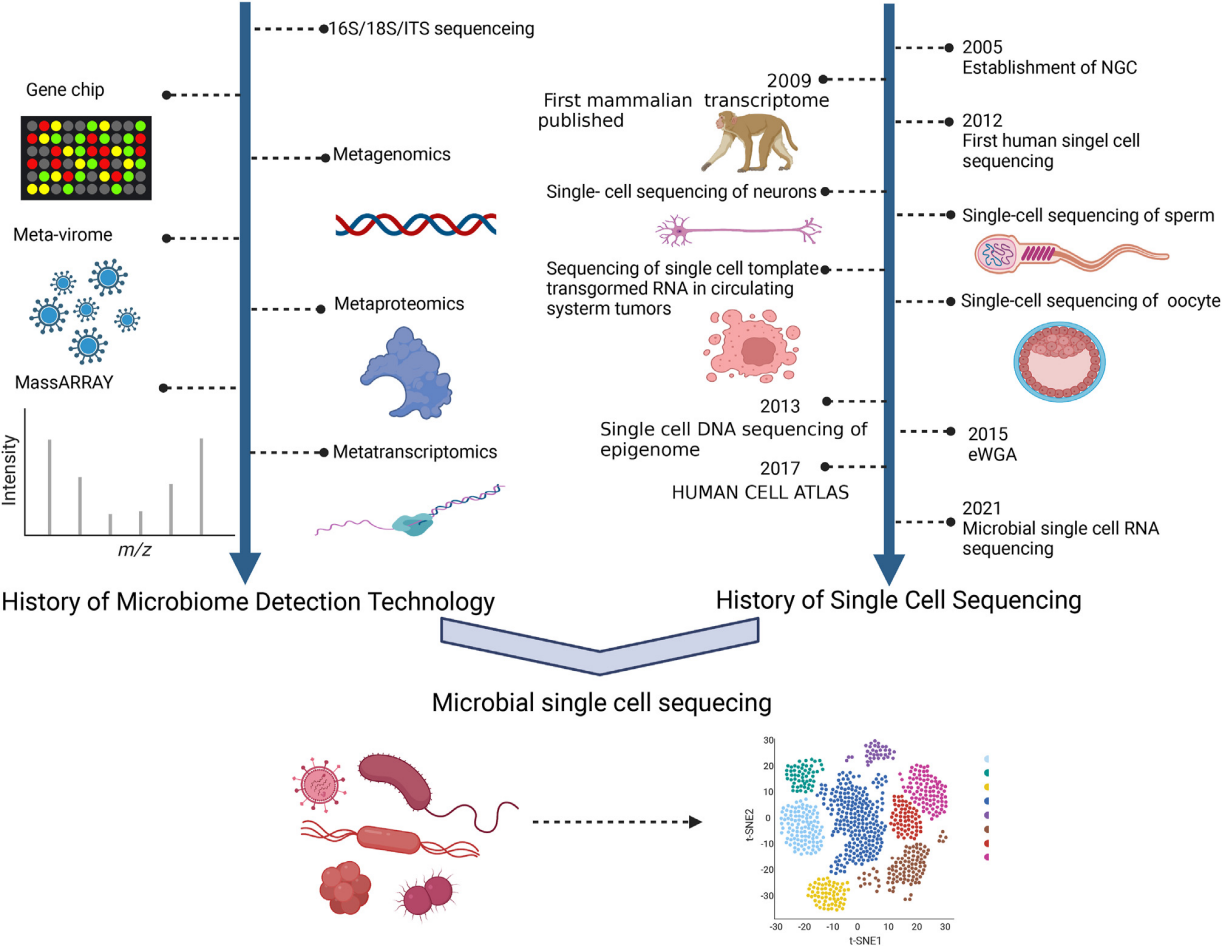
History of single-cell sequencing technology in microbiome. The timeline on the left lists the important events in the development of microbiome detection technology, while the timeline on the right lists the important events in the development of single-cell sequencing technology.

In recent years, microbial sequencing techniques have developed rapidly, and their development process is shown in Figure 1.

## The history of microbial sequencing technology

Microbes are closely related to human health. In the 19th and early 20th centuries, microbiology was firmly established and increasingly integrated with epidemiology, immunology, and genetics.^22^, ^23^, ^24^ Microbial detection technology was also gradually developed and improved for research. Sequencing technology has come to the third generation. The first human genome map was completed in 2001 and represented by the Sanger method and the dideoxy chain termination method.^25^ Compared with the first generation sequencing, the second generation sequencing developed towards the direction of high throughput and low cost,^26^ including Illumina Solexa synthetic sequencing,^27^ Roche 454 pyrosequencing,^28^ ABI SOLiD linkage sequencing,^29^ and BGI nanosphere sequencing,^30^ which breaks through the problem of sequencing volume. The gene fragments to be tested were divided into small fragments, and a large number of sequences were sequenced through the integration of overlapping regions at one time.^31^ The third-generation sequencing includes nanopore sequencing from Oxford Nanopore^32^ and single molecule targeted sequencing from Pacific Bioscience.^33^ The third generation sequencing is an upgrade of second-generation sequencing. The length of sequencing is increased to about 10 kb, and direct sequencing can be performed without PCR enrichment.^34^

## The development history of microbial sequencing

Amplification sequencing refers to the process of extracting the DNA of the organism, amplifying the variable region or full length of 16S rDNA, 18SrDNA, or ITS (internal transtranscription spacer) with a universal primer, and finally sequencing it. 16S/18S/ITS variable region sequencing or full-length sequencing can be used to detect microbial diversity. In the 1970s, researchers discovered that certain sequences of ribosomal RNA (rRNA) could be useful for the study of microbial phylogeny and genetic differences among microbial species. rRNA sequences are conserved in organisms of the same genus and species but differ significantly among species. According to the sedimentation coefficient, bacterial rRNA can be divided into three types, namely 5S, 16S, and 23S rRNA. 16S rDNA is the DNA sequence encoding rRNA on bacterial chromosomes, which exists in all bacterial chromosome genes and can reflect the differences between different bacterial genera. 16S rDNA has a moderate size, about 1.5 kb, and is highly conservative in structure and function. Meanwhile, it is known as a “bacterial fossil” that can not only reflect the differences between different bacterial genera but also obtain its sequence easily by sequencing technology.^35^ 16S rDNA sequencing can be used to analyze the structural diversity of the bacterial community and reveal the active or recently active members of the bacterial community.^36^ Currently, it is widely used for the detection and identification of pathogenic bacteria. Universal primers are used to amplify and sequence the 16S rRNA conserved region. With the Basic Local Alignment Search Tool for analysis of nucleic acid sequences in public nucleic acid libraries, biological information, such as the species category of bacteria, can be determined.^37^ 18S rDNA, the DNA sequence encoding the rRNA of the small subunit of the eukaryotic ribosome, is divided into the conserved region and highly variable region in structure. The conserved region reflects the genetic relationship between biological species, while the highly variable region reflects the differences between species. Full-length sequencing of the highly variable can be used to analyze the community structure of eukaryotic microorganisms. ITS is a transcription interval sequence of 18S rDNA and 28S rDNA in eukaryotes. In eukaryotes, ribosomal DNA is composed of ribosomal genes and their adjacent spacer regions (18S gene/ITS1, 5.8S gene/ITS2, 28S gene/intergenic spacer) a, and its genome sequence from 5′ to 3′ is an external transcribed spacer. ITS1 and ITS2, as non-coding regions, bear little selection pressure, with great relative changes, and can provide heritable traits for detailed systematic analysis. ITS sequencing was applied to the analysis of fungal community structure.^38^ However, 16S/18S/ITS sequencing also has some limitations, such as the inability to distinguish the high interspecific similarity between two species, the possible heterogeneity of gene sequences, and the depth bias, which affects the abundance and diversity of microbial communities.^12^^,^^39^

Metagenomic sequencing takes the DNA of all the genomic genetic material of bacteria, fungi, archaea, and viruses in the environment as the research object. The metagenomic method is the study object of all the genome genetic material DNA of all bacteria, fungi, and archaea in the environment. The whole DNA is assembled *de novo*, and genes are used as research units to study species diversity, gene structure, differential genes, functional genes, *etc.*, and can be applied to the development of new physiological active substances such as biological enzymes.^39^^,^^40^ Meanwhile, it provides a new approach to the study of the genetic and metabolic diversity of microbial communities.^39^^,^^41^ Macro transcriptome sequencing takes all RNA in the environment as the object, which is often complementary to metagenome, and it can reveal the changes in complex microbial communities and effectively expand the utilization space of microbial resources for transcriptional profiling. With the assistance of transcription profiles obtained from the intestinal microbial community, it is possible to correlate the expressed genes of the microbial community with human metabolism and physiological phenotypes.^42^ Moreover, macrotranscriptome is a satisfactory method to measure the composition of active strains and the expression of active genes in a specific microbial community in a specific environment. Combined with the detection of physical and chemical factors, macrotranscriptome can investigate the differences in the composition of active components between different microbial communities in time and space caused by differences in physical and chemical indexes.^43^ Meta proteomics sequencing is a new technology that uses protein technology to explore and analyze all proteins produced by specific microbial communities on a large scale, with powerful functions in the fields of gene expression in extreme environments, development of special functional proteins, and ecological element cycle.^44^ Metagenome-wide association study, similar to genome-wide association study, identifies metagenomic marker units related to traits through metagenomic scale analysis,^45^ and their main application direction is to discover microorganisms or gene markers related to diseases or other host phenotypic traits through intestinal flora mining, to diagnose host diseases and further analyze their molecular mechanisms, thus providing feasible solutions for disease treatment.

According to the flight time of ions with different mass/charge ratios to the detector, nucleic acid mass spectrometry technology ionizes nucleic acid to distinguish the different molecular weights in different samples, thus completing the detection of genes. Nucleic acid mass spectrometry is the application of matrix-assisted laser desorption ionization time-of-flight mass spectrometry which is a composite technology combining multiple PCR reactions with mass spectrometry^46^ at the nucleic acid level. It was initially used for microbial identification of protein levels. Its application at the nucleic acid level has a short development time, and its main application field is single nucleotide polymorphism genotyping.^47^ Firstly, the genome fragment containing single nucleotide polymorphism was amplified by PCR, after which the precise molecular weight of the sample analyte was obtained by detecting the flight time of nucleic acid molecules in the vacuum tube, to detect the single nucleotide polymorphism site information.^48^ The principle of nucleic acid mass spectrometry microbial detection is to use matrix-assisted laser desorption ionization time-of-flight mass spectrometry to detect the products of multiple PCR reactions, namely the mass size of the products of single base extension, to determine the presence or absence of target genes, to further determine the presence or absence of target pathogens in the samples.^49^

Microarray techniques sequence a large amount of DNA or RNA at once by attaching a large number of probes to a support and hybridizing them with a labeled sample. In the 1980s and early 1990s, Stephen P.A. Fodor et al studied how to combine biological data with semiconductor technology and tried to establish a large amount of biological data on small glass chips. In 1991, they made the world's first oligonucleotide chip by synthesizing oligonucleotide fragments *in situ* on a glass sheet of about 1 cm^2^. In 1997, the world's first whole genome chip, namely a yeast whole genome chip containing 6166 genes, was completed at Brown Laboratory of Stanford University. Existing chip preparation techniques include *in situ* photolithography synthesis of oligonucleotides, Ink-Jet printing synthesis, and spotting samples. In the field of microbiology, gene chip technology has been used in the study of microbial functional genomics, biological pathogenic mechanisms, infectious disease diagnosis, environmental microbial detection, *etc*.^50^, ^51^, ^52^ For example, gene chip technology that helps improve the diagnostic efficiency of the detection of drug-resistant *Mycobacterium tuberculosis* in clinical sputum samples could be directly used in the diagnosis of drug-resistant tuberculosis.^53^

With the deepening of microbiome research, people gradually understand the close relationship between microorganisms and human health, such as how microorganisms mediate digestion and disease processes to discover new ideas related to cancer and depression.^54^^,^^55^

## The development history of single-cell sequencing

SCS techniques refer to the amplification and sequencing of the transcriptome or genome at the level of a single cell to detect data from multiple omics, including genomics, transcriptomics, epigenomics, and proteomics. SCS technology mainly involves single-cell genome sequencing, single-cell transcriptome sequencing, and single-cell epigenome sequencing.^56^ Due to its detection at the single-cell resolution level, SCS technology has strong application potential. For example, although nanomaterials have potential toxicity at the cellular level, they have been widely used in biomedicine.^57^ Three-dimensional tissue engineering has entered a new stage of combining medical tissue engineering product production with nanotechnology and achieved initial results in the treatment of a variety of diseases, such as neurological diseases, while the current bottleneck is the rapid loss of cell differentiation function.^58^, ^59^, ^60^ Considering the heterogeneity of cells, SCS technology can be committed to the comparison of genomic data of cultured cells and normal cells. Comparison of genomic data at the single-cell level between the two groups of cells is conducive to providing data reference for genetic engineering technology in solving the poor differentiation ability of cultured cells. SCS technology has been widely used in eukaryotes, and its development process is displayed in Figure 1.

In 2009, Tang et al completed the first transcriptome sequencing of mammalian single-cell RNA and developed the first single-cell transcriptome sequencing technology, marking the arrival of the era of SCS.^61^ In 2011, Nicholas et al developed single-cell genome sequencing technology.^62^ The first high-throughput sequencing of single-cell DNA could break through the difficulties of traditional cancer genome research that could not judge the source and frequency of specific mutations, the occurrence and evolution of cancer, and active and passive mutation identification. In 2013, single-cell whole-genome DNA methylation detection was developed.^63^ Subsequently, scientists focused on cell sorting technology, nucleic acid amplification technology, signal-to-noise ratio improvement, and other aspects of continuous optimization and improvement, but also further pioneered single-cell Hi-C, single-cell ChIP-seq, and single-cell ATAC-seq technology. For example, Xie Xiaoliang's team invented the DNA amplification technology MALBAC to improve the bias of PCR amplification, reduce genome complexity, and improve sequencing accuracy.^64^ However, this DNA amplification technology has two obvious drawbacks: misamplification of the preferred polymerase can produce thousands of false positives on the genome; the short sequence read length contains almost no haploid type information. Early methods of single-cell extraction mainly include limited dilution, flow separation, laser cutting, and micromanipulation. However, there are many drawbacks: the common disadvantage is the high cost of capture; the flow separation method is prone to errors and is not suitable for micro-samples; the laser cutting method is complicated to operate; the cell flux of microdissection is low.^65^

Until 2014, the flux of single-cell technology remained below 100 cells. With the continuous updating and iteration of sequencing technology, SCS gradually realized the transformation from low- to high-throughput detection. From 2014 to 2015, techniques, such as microfluid-based microdroplet binding single cells (CytoSeq, Drop-Seq, InDrop, *etc*.), microporous array-based single cell separation (BD Rhapsody, Singleron, *etc*.), and split-pool ligation-based transcriptome sequencing increased the fluxes to the order of 10,000–100,000 cells. Moreover, to accurately detect genomic variation, emulsion whole-genome amplification, a combination of “emulsion + multiple displacement amplification + microfluidic chip” improved amplification uniformity, genomic coverage, and replication accuracy.^66^ With further improvement, single-stranded sequencing using microfluidic reactors technology based on microfluidic reactor could be used for precise single-cell genome sequencing and haploid typing.^67^ In 2017, Guo's team took the lead in developing the whole genome sequencing technology for simultaneous chromatin status, DNA methylation, genome copy number variation, and chromosome ploidy of a single cell.^68^

The official publication of the Human Cell Atlas project in 2017 is an important milestone in the industrialization of high-throughput single-cell research. During 2017–2018, 10X Genomics (combining Barcoding and Microfluidics) and BD Rhapsody technology (microplate) made SCS widely available. Since then, SCS technology has developed rapidly. In 2021, Wang's team developed single-cell multiomics sequencing technology (scNOMeRe-seq) that can simultaneously analyze the RNA expression, DNA methylation, and chromatin accessibility of a single cell. A comprehensive joint analysis of the regulatory relationships at multiple levels in the single-cell resolution of mammalian preimplantation embryos was conducted for the first time.^69^

With the maturation of single-cell sorting and amplification technologies, SCS can solve the problem of cell heterogeneity and play an important role in neurobiology, reproductive genetics, organogenesis, cancer biology, medical diagnosis, immunology, microbiology, tissue chimerism, embryology, prenatal diagnosis, *etc*.^70^, ^71^, ^72^, ^73^, ^74^ For example, through scRNA sequence analysis of immune and stromal populations in colorectal cancer patients, it was determined that specific macrophages and conventional dendritic cell subsets were key mediators of intercellular cross-talk in the tumor microenvironment. Finally, the key cellular role in regulating tumor immunity was determined.^75^ Disease heterogeneity among refractory multiple myeloma patients was analyzed by scRNA-seq, which provided new targets for multiple myeloma resistance and drug therapy response.^76^^,^^77^

## The generation of microbial single-cell sequencing technology

Microorganisms are the most suitable research projects for single-cell genome sequencing. It is estimated that the vast majority of microbes (99% of all species) cannot be cultured. The culturable microbes are known as the “dark matter” of biology. While metagenomic approaches contribute to the understanding of the genetic makeup of this complex environment, the relationship between species and genes remains unknown. Therefore, only with the application of single-cell genome technology can unicellular organisms understand the relationship between their genome functions.

Recent microbiome studies have found that many microbiome-related clinical markers are not phenotypes caused by the microbiome, and studies on the interaction among microbial individuals, microbe-host interactions, and multidimensional molecular mechanisms of these combinations require us to find a microbial detection method that can be accurate to the strain level.^78^ What's more, intra-species heterogeneity determines the survival of the entire microbial community to some extent. For example, changes in the single locus of *Bacillus subtilis* biofilm formation phenotype directly alter community structure to the extent that the loss of apex predators is comparable.^79^ *Staphylococcus aureus* can infect almost every host organ due to cell heterogeneity that could produce multiple infection modes, such as acute bacteremia and endocarditis.^80^

However, microbial metagenomics cannot efficiently assign DNA sequences common to multiple taxa in a single sample. For instance, when a species has multiple strains or when homologous sequences appear in the genome of multiple taxa. As SCS technology has been widely used in eukaryotes such as animals and plants, it has gradually touched the field of microorganisms. Single-cell microbial sequencing took shape in 2016.^81^ Microbial split-pool ligation transcriptomics technology developed by Georg Seelig et al in 2021 achieved single-cell transcriptome sequencing of *Bacillus subtilis*, which revealed the heterogeneous phenomena of bacterial behavior, metabolism, and stress response in very few people.^82^ Microbial split-pool ligation transcriptomics technology enables the clustering of bacteria and the discovery of different cell subpopulations (gene expression that exhibits different metabolic, stress response, or developmental pathways). Bulk RNA-seq and low-throughput SCS methods are not available to uncover rare bacterial cell states that are physiologically relevant. However, microbial split-pool ligation transcriptomics could detect rare randomly induced developmental states, such as bacteria with horizontal gene transfer (HGT) events. Besides, tRNA, microRNA, and lncRNA can be detected.

In 2022, Zheng et al invented Microbe-seq technology that solved the challenge of genome sequencing at the microbial strain level and improved the limited throughput of microbial strain sequencing due to microplate-based SCS. Microbe-seq technology integrates a variety of dropper microfluidic manipulation techniques with custom-developed bioinformatics analysis tools to obtain genomic information from thousands of single-celled microorganisms from complex microbial communities without the need for culture and assemble high-quality strain-level genomes. Thus, the genomes of microbial communities can be explored without the loss of resolution or applicability to a wide range of species.^83^

## The technologies of microbial SCS

### Microbial single-cell sorting technology

**Figure 2:**
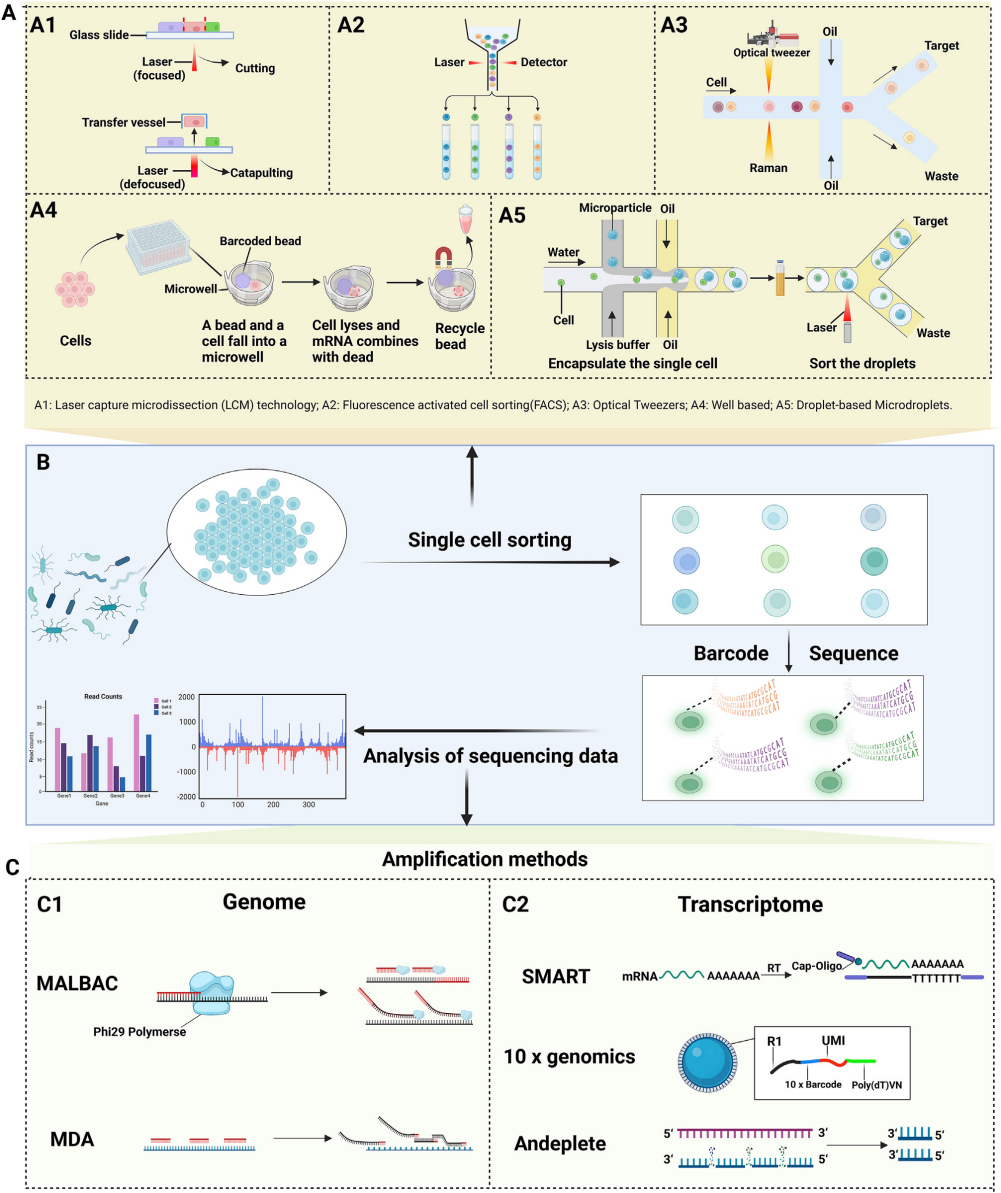
Principles of microbial single-cell sequencing. **(A)** Five common methods for single cell isolation of microorganisms. **(B)** The basic process of microbial single-cell sequencing. **(C)** Principles of microbial single-cell genome sequencing and transcriptome sequencing.

Microbial single-cell isolation is the key to the success of SCS. Before SCS, a single cell needs to be isolated first. Different types of SCS techniques may use different cell isolation techniques. Generally speaking, there are mainly five types of cell separation techniques, as shown in Figure 2.

### Laser capture microdissection technology

Laser capture microdissection is used to obtain target cells without damaging the structure and morphology of tissues and precisely separate a single cell from tissues.^84^ At present, it has been applied in the sorting of microbial cells.^85^, ^86^, ^87^ The laser capture microdissection system includes an inverted microscope, a solid-state infrared laser diode, a laser control device, a joystick that controls the microscope's loading platform, an electrically coupled camera, and a color display. The procedure is described as follows. The prepared tissue sections were fixed by a vacuum pump in the loading platform of the inverted microscope. Then, the inverted microscope mode was selected according to the specimen, the microscope was adjusted, and the target area was located. A collection cap with ethylene vinyl acetate film is placed on the target tissue or cell and a low-energy near-infrared laser was applied to irradiate the bottom of the film to soften the film and generate adhesive force. The target tissue or cell was attached to the film, to separate it from the surrounding tissue or cell. This film was thermoplastic, with a thickness of about 100∼200 μm, and a maximum absorption peak of close to the infrared laser wavelength. It can absorb most of the energy that is generated by the laser and instantly raise the temperature of the laser beam irradiation area to 90 °C. Afterward, a few milliseconds were maintained after cooling rapidly to ensure that biological macromolecules were not damaged.^88^ The cut tissues or cells were transferred to a centrifuge tube to extract DNA, RNA, or protein for downstream analysis.

### Fluorescence-activated cell sorting

Fluorescence-activated cell sorting is a technology for sorting individual or group cells according to cell-specific molecular markers or cell light scattering characteristics by flow cytometry. It is currently the most commonly used single-cell classification technology, and it uses a laser beam to excite single-line flowing cells bound to fluorescein-labeled antibodies and sorts the cells based on the excited fluorescence signal. Different physicochemical or optical properties of cells were scattered by laser light, which can usually be enhanced by synthetic substances such as fluorescent dyes. After fluorescent staining, cell samples were driven by air pressure into the flow chamber of sheath fluid (balanced electrolyte solution without fluorescent background) and then ejected, after which the flow was stirred by ultrasonic oscillation to break the flow into a series of uniform droplets containing at most one cell. After the cell was irradiated by the laser, the fluorescence and scattered light signals were collected and analyzed by the downstream optical system. If the signal of the sample cell was consistent with the characteristics of the target cell, it would be selectively induced by positive, negative, or no charge instantaneously when the droplet was just formed, thus deflecting under the action of the electromagnetic field and entering the corresponding collection tube.^89^, ^90^, ^91^

### Optical tweezers

Optical tweezers technology utilizes the interaction between the single-beam gradient force trap of the focused laser beam and the particle to grasp and control the particle.^92^ The application of optical tweezers to microbial separation depends on microfluidic technology, combined with Raman spectroscopy with single-cell optical tweezers, such as Raman spectroscopy with single-cell optical tweezers. The microfluidic method controls the flow of microbial samples in the chip and conducts Raman detection after being captured by optical tweezers. When the detection is the target sample, it is sorted through optical tweezers to the channel in the collection area, while the non-target sample is released and flowed into the waste liquid area.^93^^,^^94^ Based on this principle, a Raman-activated gravity-driven single-cell encapsulation and sequencing for the microbial single cell in a liquid phase environment was developed by Xu et al by combining optical tweezers and microfluidic droplet technology. Bacterial single cells with specific Raman phenotypes can be precisely isolated from the population. The Raman sorting process of “single cell–single droplet–single tube” can be realized quickly, accurately, and simply, and it was directly coupled with downstream cell culture or genome analysis.^95^ Ge et al have developed and stimulated Raman scattering-two-photon fluorescence *in situ* hybridization, which achieved imaging speeds of individual microbe cells within 10–100 ms. It is two to three orders of magnitude and faster than the current methods. Combined with deuterium in heavy water as an activity marker, the metabolic responses of 30,000 single cells in the human intestinal microbiome to various mucosal sugars were described, and it was found that intestinal mucosal glucose metabolism was usually dominated by *Bacteroidetes*.^96^

### Microwell-based single-cell separation

Based on the classical microwell-based single-cell separation, 100–40,000 cells can be separated in a single experiment.^97^ A cell was transferred to a single hole in the porous plate to serve as a discrete reaction vessel for subsequent steps. A micropore (honeycomb plate) consists of one single cell and one bead. The cell suspensions were injected through the injection holes and naturally sank into the reaction holes. Subsequently, the beads were then injected through the injection holes to capture the cells in a single reaction hole. Sequence structures in beads captured the poly-A tails of the free mRNA. Finally, the excess gel microbeads can be washed away to achieve the purpose of single-cell capture.^98^, ^99^, ^100^

As a single-cell sorting technique based on combined barcode label RNA, split-pool ligation-based transcriptome sequencing is an upgrade of the microporous method. This technique eliminated the need to separate individual cells that could be used as their own RNA “isolation chamber” and distributed into a 96-well plate during the first round of barcoding, and the cell pool can be divided into groups. Intracellular reverse transcription was repeated many times to introduce a unique barcode sequence for each cell's RNA using specific barcode primers added to their RNA.^101^ Microbial split-pool ligation transcriptomics is a high-throughput method for scRNA-seq using split-pool barcode technology. The prerequisite for the isolation of microbial single-cell RNA was to use lysozyme and mild detergent Tween-20 to crack microbial cell walls, add a poly-A tail to mRNA through PAP enzyme, and then introduce the combined bar code for intracellular RNA three times. After *in situ* reverse transcription and two intracellular ligations, unique labeled cDNA was obtained, and finally, cell lysis and daughter library generation were performed.^82^

### Droplet-based method

The droplet method mainly uses droplet microfluidic technology that can be upgraded from a small number of droplets to thousands of droplets and is a high-throughput analytical tool. Common droplet microfluidic systems mainly include three parts, namely a micro-injection pump, a microfluidic chip, and a high-throughput screening system. Generating microdroplets with uniform size and stable velocity is the key to the normal operation of the system. As the power source of the whole system, the micro-injection pump provides stable microliquid flow and stable and fine power for the production of microdroplets. The key point of droplet microfluidic technology is to use two incompatible liquids as the continuous phase and discrete phase^102^: one is usually an organic liquid (oil) and wets the channel walls; the other is usually the water phase and is split into droplets. Single cells were wrapped in oil emulsion droplets that were dispersed into small droplets containing single cells, each of which served as a closed reaction container for single-cell encapsulation. Microtubule structure and two-phase flow rate ratio controlled droplet formation.^103^^,^^104^ The continuous phase and discrete phase entered different microtubule channels under the driving pump with a fixed volume flow rate. When two-phase fluids met at the intersection, the discrete phase was squeezed and sheared by the continuous phase to generate discontinuous droplets that were dispersed in the continuous phase.^105^, ^106^, ^107^ To avoid the damage of voltage to microbial cells, the current was insulated, so the water phase was a discrete phase and the oil phase was a continuous phase.^108^ There were microbial cells in the discrete phase fluid. In addition, according to the size of the isolated single cell, the size of the droplet can be adjusted by the two-phase flow rate ratio and up to the range of even soaring. Therefore, the isolation of single cells was completed. However, due to the instability of the droplet, there may be a droplet empty load in the single-cell separation step or two or even more cells in one droplet due to improper size regulation.^109^

Microfluidic cells were lysed and labeled by droplet method using four microfluidic devices. The droplet used in the first microfluidic device contained a cracking reagent that was manipulated so that thousands of microbes were individually wrapped in the droplet to break apart the microbes and release their DNA. The droplets used in the second microfluidic device contained amplification reagents to amplify individual microbial DNA. In the third microfluidic device, the amplified DNA was segmented and the splice joint sequence was added. In the fourth microfluidic device, PCR reagents and microspheres with DNA barcodes were added to the droplet, and barcode primers were connected to fragments of DNA molecules by PCR to complete genome labeling. Finally, all the DNA in the droplet was combined for database sequencing. A collection of sequencing sequences with the same barcode, known as a single amplified genome, was obtained by the droplet method. Due to the uniqueness of each droplet barcode sequence, the genome sequence can be decomposed and reduced to the level of a single microorganism through identification tags.^83^

## The technologies of labeling and sequencing

### Microbial single-cell genome amplification

Currently, there are two main methods for microbial single-cell genome amplification, namely multiple annealing and looping-based amplification cycles (MALBAC) and multiple displacement amplification.

Almost all single-celled microorganisms have been sequenced using the same whole-genome amplification reaction. This technique is one of the isothermal amplification techniques. If random primers are used, the main dependence is to perform chain substitution amplification with Phi29DNA polymerase and random hexamer, so that the double-stranded DNA can be unchained, after which the original template can be greatly amplified at room temperature.^110^^,^^111^

The principle of MALBAC is to amplify a small amount of whole genome DNA from isolated individual cells, obtain a complete genome with high coverage, and then capture the genome through exon for high-throughput sequencing. The amplification primers designed by MALBAC have eight random sequences, and the bases on the random sequences can be randomly hybridized to the complementary sequences of genomic DNA. A new DNA strand can be made by using the enzyme Phi 29 DNA polymerase. The enzyme Phi 29 DNA polymerase can also unstrand strands of synthesized DNA to form new strands, which could eventually increase the number of new strands of DNA that can be made per cycle by several to a hundred times. MALBAC amplification mainly goes through five MALBAC cycles. The first loop yielded a fragment of DNA with a common sequence at the 5′ end. After the second cycle, most of the amplification products were the 5′ end with the general sequence and the 3′ end with the complementary sequence fragments. At the end of each cycle, a step of 58 °C annealing was added. This annealing process allows both ends of the fully expanded product to undergo intra-chain hybridization, thus avoiding the self-exponential expansion of the 3′ end of the fully expanded product.^64^^,^^112^

### Microbial single-cell transcriptome amplification

There are two main methods for microbial single-cell transcriptome amplification, namely SMART (Simple Method for Amplifying RNA Targets) amplification and AnyDeplete.

SMART amplification technology can be used for sequencing single cell mRNA of microorganisms. The core technology is the design of two special primers. Moloney murine leukemia virus reverse transcriptase was used for reverse transcription. The 3′-terminal SMART primer with oligo (dG) was added in advance in the reaction of synthesizing cDNA. Since reverse transcriptase synthesizes cDNA using mRNA as a template when reaching the 5′-terminal of mRNA, it continuously added several dC to the synthesized cDNA ends when it met the unique “cap structure” of eukaryotic mRNA, that was methylated G. The oligo (dG) of the SMART primer paired with several Cs protruding from the synthetic cDNA end to form the extension template of cDNA. The reverse transcriptase automatically converted the template and continued to extend the single strand of cDNA to the end of the primer using the SMART primer as the extension template. All cDNA single strands obtained in this way had a starting primer sequence containing oligo (dT) at one end and a known SMART primer sequence at the other end. After synthesizing the second strand, it can be amplified with a universal primer. Since only mRNA with a 5′ cap structure can be amplified by this reaction, the amplified cDNA was a full-length cDNA. The main information obtained by SMART technology was mRNA information, and most lncRNA information could be lost.^113^^,^^114^

AnyDeplete technology can be used for sequencing mRNA and lncRNA of microbial single cells. First, random primers were used for one-strand synthesis, with the one-strand synthesis of introduced nucleotide analogues for enzyme cleavage interruption, and two-strand synthesis also introduced nucleotide analogues for chain specificity. Afterward, a joint was added to both ends, and a chain with a nucleotide analogue was used for enzymatic digestion. When the single-strand library was formed, the specific primers were designed to bind to the rRNA library, and the rRNA library was extended by annealing. There are specific restriction sites on the reverse adaptor. When a double-stranded structure was formed, the restriction sites were identified and the joints were cut off. In this way, libraries formed by rRNA did not have complete joints, while other libraries had complete joints. Anydeplete technology, such as the 10× genomics technology, contains molecular labels and can analyze duplication and PCR mutation sites. Anydeplete technology can be used for degradation samples to ensure the integrity of the 5′ end and 3′ end information, and to obtain mRNA and lncRNA information at the same time.

### Characteristics of Microbe-seq

**Figure 3:**
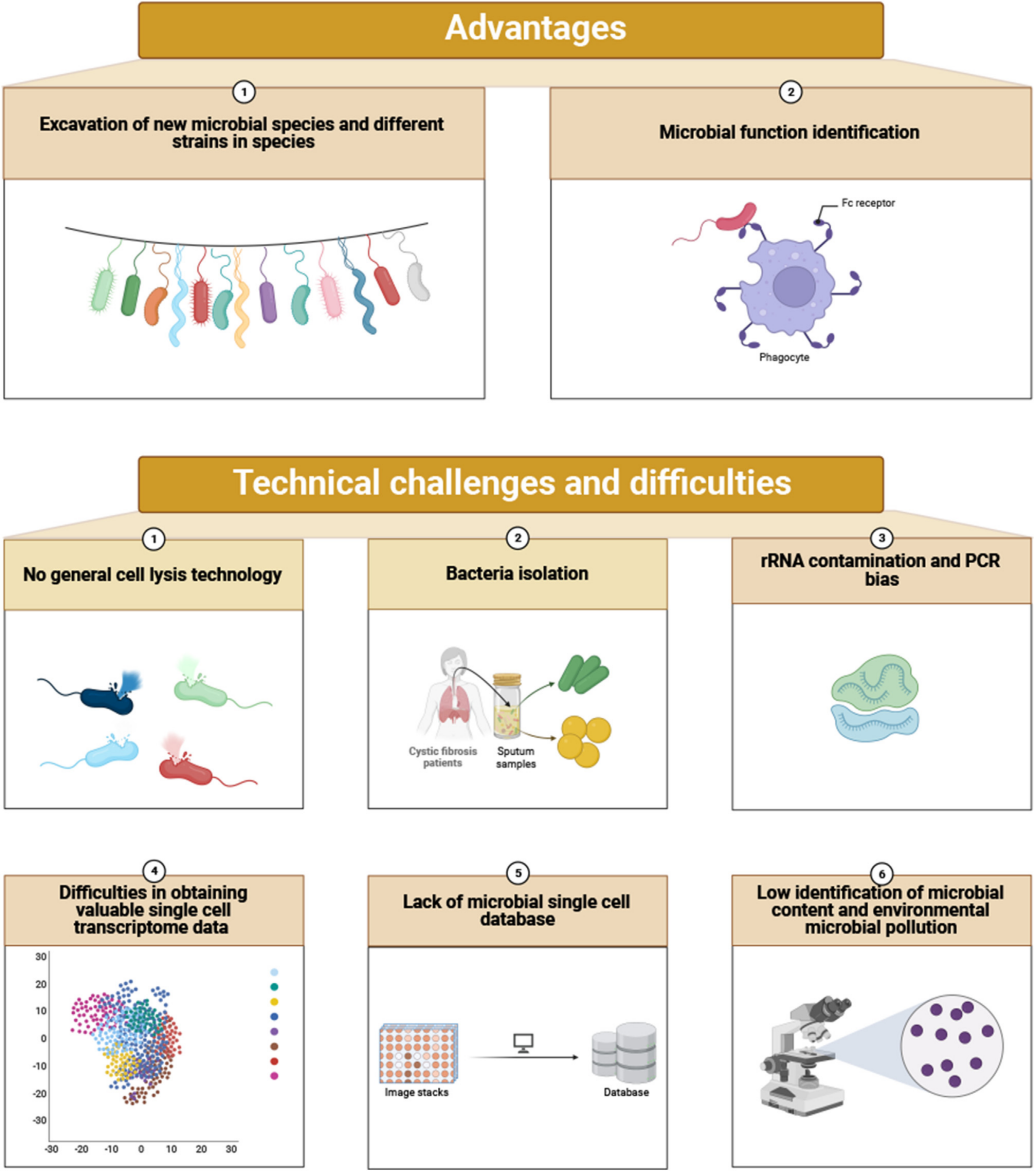
Characteristics and advantages/disadvantages of microbial single-cell sequencing.

Before the emergence of Microbe-seq technology, the main methods of microbial sequencing were 16S amplicon sequencing and microbial genome sequencing, while the above two methods had obvious defects. The reference sequence database is incomplete, and the quality of sample DNA is high. The genomic information cannot be analyzed at the level of a single bacterium, and it is difficult to find the potential strains that have not been cultured. Only the taxonomic groups with known genetic markers rich in taxonomic information can be amplified and analyzed,^81^^,^^115^ which greatly limits the analysis of the resolution of the genome of individual bacteria from the same community, and limits the understanding of the composition and structure of the microbiome and its genome (Fig. 3).

### Excavation of new microbial species and different strains in species

Microbe-seq has a high taxonomic resolution and can be used for individual gene detection in response to bacterial heterogeneity. The advantage of microbial SCS is that the heterogeneity of single cells can be avoided by homogenization of mixed (Bulk) samples. Zheng et al sequenced seven intestinal microbiome samples from a single individual using Microbe-seq, and obtained the genome information of 21,914 single-celled microorganisms, from which 76 species-level genomes, including a variety of bacteria that were difficult to culture, were assembled, and multiple strains of 10 species were found.^83^ Based on the detection of strain level, Microbe-seq can observe the different metabolic levels, stress response, and gene expression of strains in the same community, to find the potential genome without culture.^116^^,^^117^ Marcy et al revealed the genetic information of rare bacteria that could not be cultured in the human mouth through single-cell microbial sequencing, especially members of the TM7 phylum.^118^ Microbe-seq can simultaneously detect small fragment copy number variation and high-precision single nucleotide polymorphism in microbial single cells.^66^ Microbial SCS uses single-cell resolution to evaluate microbial genome abundance and population diversity to reduce the impacts of amplification bias and copy number variation on genome sequencing results.^119^

### Microbial function identification

Microbe-seq can be used to analyze the functional genes, metabolic pathways, and their interactions with the host. Swan et al isolated and sequenced microbial single cells in marine samples and obtained the whole genome information of *Deltaproteobacteria cluster SAR324*, *Gammaproteobacteria clusters ARCTIC96BD-19*, *Agg47*, *Oceanospirillales*, and other strains, which revealed the important role of uncultured *Proteobacteria* lineages in the marine carbon cycle.^120^ Microorganisms can obtain external DNA through HGT, a mechanism that enables microorganisms to rapidly adapt to environmental changes, provides a competitive advantage for the organism, and may change the relationship between it and the host.^121^ Microbe-seq can detect horizontal transfer of microbial genomic mobile genetic elements (MGEs), including plasmids, phages, and conjugated transposons (CTns), which was not possible with previous amplicon sequencing and metagenomic sequencing techniques. Lawrence et al combined fluorescence-activated cell sorting with multiple displacement amplification technology, and the single-cell genome of human intestinal microorganisms was analyzed for feature analysis. It proved the feasibility of SCS for microbial-level gene transfer and the mechanism of interaction with bacterial host genome.^122^^,^^123^ Moreover, Microbe-seq can conduct cell clustering by detecting individual genotype differences and find rare cell subsets and randomly induced developmental states. Through microbial SCS, Kuchina et al not only excavated *Bacillus subtilis* with HGT event but also found that *Bacillus subtilis* cells could take up extracellular DNA and integrate it into chromosomes through random instantaneous differentiation into a state of natural ability, which improved the survival rate under adverse conditions.^82^^,^^122^ Chijiiwa et al used the single amplified genome-gel platform to detect bacterial genomes in the feces of inulin-fed mice and obtained 346 single amplified genomes. A newly discovered species of bacteroides was obtained from the study, and a specific gene cluster for inulin decomposition and a specific metabolic pathway for the production of specific short-chain fatty acids were identified.^124^ Therefore, single-cell microbial sequencing can be applied to characterize uncultured bacteria with specific functions in the microbiome and estimate their metabolic characteristics.

### Technical challenges and difficulties

However, there are still some technical difficulties in the application of SCS in microbial genome or transcriptome sequencing, thus resulting in slow progress in the application of microbiome sequencing. The difficulties summarized are related to the structure and genes of microorganisms themselves.

Firstly, the composition of microbial cytoderm is complex and difficult to lysis, and there is no one general cell lysis technology. Both genome sequencing and transcriptome sequencing must go through the cell wall lysis process. The existing methods of enzyme lysis of microorganisms may be biased. Microbe-seq technology is used for mixed detection of Gram-negative (*E. coli*, *Klebsiella pneumoniae*) and Gram-positive (*Staphylococcus aureus*, *Bacillus subtilis*) at similar concentrations, and the genome coverage of Gram-negative bacteria is lower than that of Gram-positive bacteria.^83^ Most microbial cell walls contain three main components including saccharides, lipids, and proteins. However, for different microorganisms, their specific components and composition ratios are different. For example, bacterial cytoderm is composed of chitin, β-glucan, and mannoglycoprotein. Due to the complex structure of the cytoderm, there is no efficient microbial single-cell lysis technique or general microbial single-cell lysis reagent.

Secondly, the isolation of bacteria in a given ecosystem is difficult. Single-cell isolation needs to overcome various problems such as bacterial self-aggregation (adhesion between the same bacterial cells)^125^ and low biomass microorganisms in the blood, mouth, nasopharynx, and vagina.^126^

Thirdly, ribosomal RNA contamination is serious and PCR bias exists. A single cell contains about 10 pg total RNA, and more than 80% of the information is rRNA. The expansion of nucleic acid from single-cell RNA to library means more than a million times. At this high expansion increment, it is difficult to avoid the problem of PCR bias.^127^^,^^128^ rRNA accounts for a large proportion, and most sequences obtained by indiscriminate reverse transcription, reamplification, and library-building sequencing are rRNA sequences, which affects the effective information and accuracy of mRNA.^82^ Therefore, new techniques are needed to correspondingly improve the efficiency of mRNA capture and targeted removal of ribosomal RNA sequences.

Fourthly, it is still challenging to mine valuable specific transcripts from the huge single-cell data. Genes use different mRNA transcripts in different tissues and cell subsets, and structural variants, such as SNV and fusion genes, are also tissue- and cell-specific. Although short-read and long-read second-generation sequencing can achieve full-length transcript coverage, it requires splicing and assembly to analyze transcript structure, with low throughput and high cost. It is still difficult to study single-cell variable shear. Long-read third-generation sequencing can obtain the full-length sequence of mRNA, and the cell subpopulation is located by cell barcode information.^129^ However, the expression matrix of about 30,000 genes can be obtained by the second-generation SCS, and the expression matrix of more than 100,000 transcripts can be obtained by the third-generation full-length sequencing. The cluster maps of the two generations of sequencing methods are very different, and the clustering often lacks specificity.

Fifthly, it is the lack of a microbial single-cell database. SCS is mainly applied to eukaryotes. The existing databases are mainly for the cell spectra of human and experimental animals, aiming to analyze the changes of cell types and functional states in different tissues and diseases of the human body at the single-cell level. At present, the database for microbial single cells is still missing.

Sixthly, it is for the identification of very low microbial content and how to eliminate the problem of microbial pollution in the environment. Microbes are in the air, in the test tube, and in the pipette head, and these microbes may affect the identification of trace microbes. Whether the current disinfection methods can completely kill all environmental microorganisms and ensure that no non-microbial RNA remains is an important problem in the determination of trace microbial DNA. By setting up more control groups, the elimination of these disturbances by bioinformatics analysis is the key to microbiological quantification.

### Application prospect of Microbe-seq

**Figure 4:**
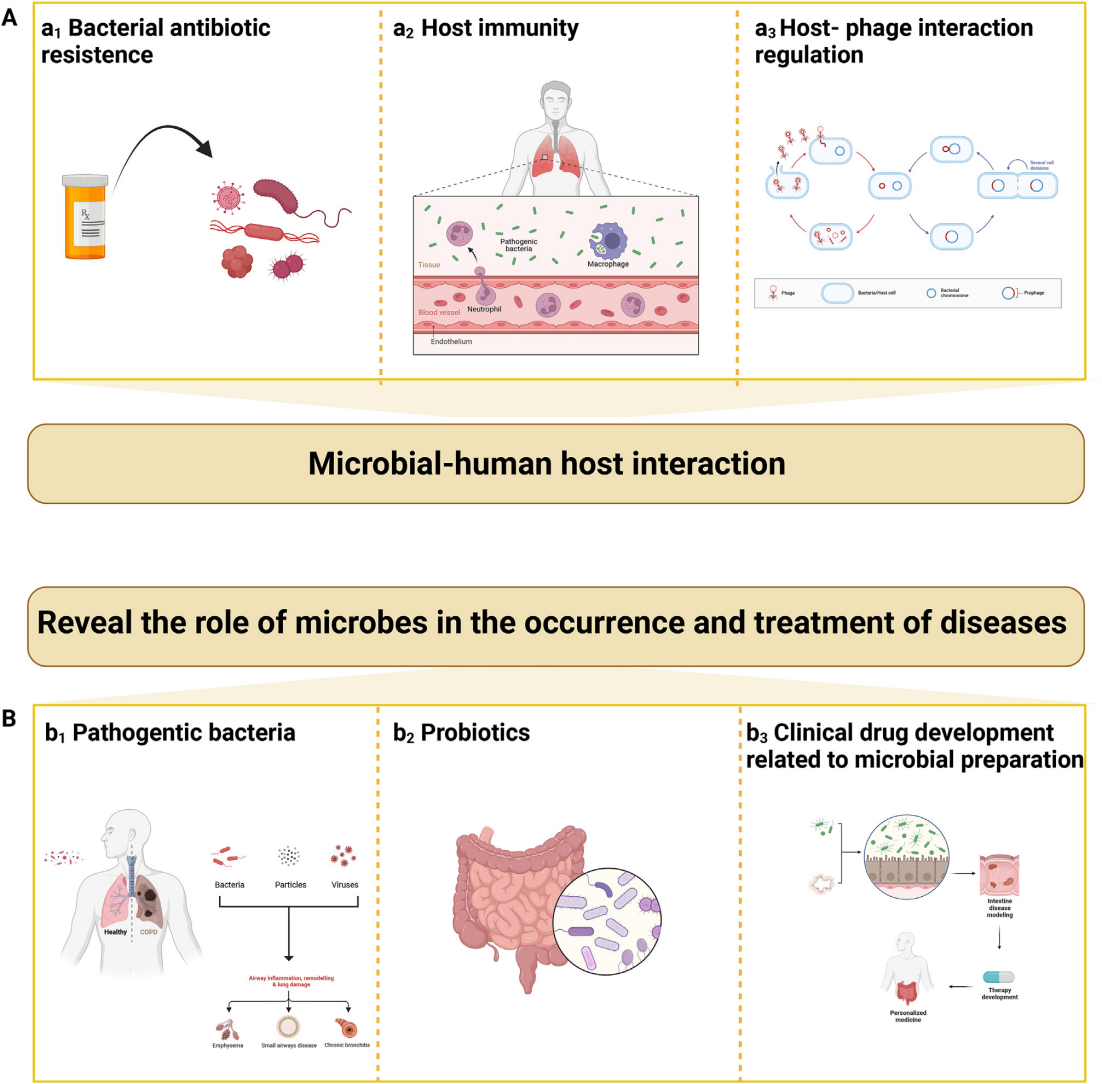
Application of microbial single-cell sequencing in microorganisms, namely in microbial individual, microorganism & host, microorganism & disease, and microorganism & environment.

Microbe-seq can analyze new microbial communities and detect microbial function at the single-cell level. The detection objects of Microbe-seq cover microbial individual, microorganism & host, microorganism & disease, and microorganism & environment, which is different from omics technology that focuses on the microbiome (Figure 4).

## Microbial-human host interaction

### Bacterial antibiotic resistance

Bacterial antibiotic resistance is divided into inherent resistance and acquired resistance. Inherent resistance is independent of antibiotic use and emerges spontaneously. For example, all *Klebsiella pneumoniae* are intrinsically resistant to ampicillin and all *Enterococcus faecalis* are resistant to cephalosporins. The acquisition of drug resistance means that under the pressure of environmental antibiotics, bacteria develop drug resistance and carry out adaptive evolution through gene mutation or acquisition of foreign DNA.^130^, ^131^, ^132^, ^133^, ^134^ From the perspective of coping mechanism, there are four main mechanisms of bacterial antibiotic resistance: (i) changing the target of antibiotic action to obtain resistance; (ii) producing one or more hydrolytic, and passivating and modifying enzymes; (iii) decreasing permeability of bacterial membranes, including bacterial biofilm formation and channel protein loss; (iv) overexpressing bacterial active efflux system. For example, *Pseudomonas aeruginosa* has a deletion of the porpoprotein gene, which restricts the entry of antibiotics into cells, and thus develops resistance to carbapenemes.^135^ The beta-lactam ring of hydrolyzed antibiotics is destroyed by Gram-negative bacteria through beta-lactamase, thus rendering it to be ineffective.^136^

Additionally, HGT is an important pathway for bacteria to acquire antibiotic resistance and pathogenicity.^133^ For example, vancomycin is an antibiotic for methicillin-resistant *Staphylococcus aureus* infections, while vancomycin-resistant *Staphylococcus aureus* can obtain the plasmid of *Enterococcus faecalis* by HGT and develop resistance.^134^ Studies have revealed that the mutation rate and HGT ratio of different strains are different.^134^^,^^137^ Previous studies on bacterial antibiotic resistance have been conducted in the form of microbiome detection, and the specific mechanism of action between the change of single cell genotype and bacterial antibiotic resistance, as well as its impacts on the flora and even the host, cannot be explored.

Microbial single-cell technology is combined with bacterial antibiotic resistance to explore the mechanism of bacterial cell-building drug tolerance from the strain level. Droplet microfluidic-driven single-cell diagnostics have been used in the diagnosis of urinary tract infections. Nearly 90% of urinary pathogens are identified with the help of PNA probe panels and droplet microcontrol technology, and 16S rRNA of single bacterial cells is quantitatively detected in a drop-supported antimicrobial sensitivity test after only 10 min of exposure to antibiotics. Pathogen detection and antimicrobial sensitivity testing could be achieved within 30 min from minimally treated urine samples.^138^ Microbe-seq is used to construct the HGT network of strains in the intestinal microbiome of a single individual, and it is found that the transfer between strains in the same phyla is significantly greater than that between strains in different phyla, which confirmes that Microbe-seq can detect HGT in multiple strains.^83^ HGT is of great significance in the study of antibiotic resistance and the pathogenicity of bacteria in human intestinal microbiota. In the clinical antimicrobial sensitivity experiment of *Helicobacter pylori*, when the levofloxacin resistance test was carried out by single-cell Raman spectroscopy, D91Y mutation was detected in HP2 and HP18 in Y027 samples. On the other hand, previous studies indicated that D91Y was included in the locus mutation of the levofloxacin resistance determination region. Besides, the AST phenotypes of the two strains detected by single-cell Raman spectroscopy were also consistent with previous studies, which verified the association between resistance genes and resistance phenotypes, and also provided a tool for direct prediction of resistance phenotypes based on single-cell genotypes.^139^

### Host immunity

The relationship between microbial and host immunity was studied at the single-cell level, and it was found that microbial variability determined the differences in host-cell responses. It has been demonstrated that the differences in cellular immune responses in the host are due to the differences in the microbes rather than the heterogeneity of host cells.^140^ For example, Avraham et al combined single-cell RNA-seq with fluorescent markers to study the response of macrophages to salmonella invasion. It was discovered that macrophage type I IFN response status was closely related to the variable PhoPQ activity of bacteria. PhoPQ is bimodal induction of type I IFN response by LPS modification on the cell surface. Host cells that internalize bacteria with high PhoPQ activity show a high type I interferon response, while host cells that internalize bacteria with low PhoPO activity display a low type I interferon response.^141^ SCS has also played an important role in COVID-19 research. Llorens-Rico et al first linked host cells to lung microbes using single-cell RNA-seq. The correlation between mycoplasma bacteria and mononuclear cell-derived macrophages was found in COVID-19 cells, which indicated that the bacteria may be phagocytic by innate immune system cells rather than attached to the surface of host cells.^142^ In addition, Abhimanyu Thakur et al included single-cell transcriptome sequencing data of 28 glioblastoma patients from a public database and found in KEGG pathway analysis that COVID-19-related genes were involved in most genes, among which C1qrs and membrane attack complex genes were up-regulated.^143^ *In vitro* cell experiments and *in vivo* experiments in mice revealed that the changes in these genes are related to the expression of endothelial cell adhesion molecules, cytokine release, and inflammatory response.^144^^,^^145^ These results supported a potential association between COVID-19 and glioblastoma and suggested that the virus could induce endothelial cell dysfunction through an inflammatory response to increase the risk of glioblastoma. Steuerman et al obtained viral information from cells by SCS of mouse lung cells infected with the influenza virus two days later, combined with vmRNA levels in the cells. They found that vmRNA is detectable in almost all cell types from 62% of epithelial cells to 22% of T cells. At the single-cell level, it was confirmed that the influenza virus not only invaded lung epithelial cells but also acted on T cells.^146^

### Host-phage interaction regulation

The study of bacteriophage and its host mechanism by Microbe-seq can provide important technical support for the application of bacteriophage for targeted therapy and bacteriophage host mechanism. Microbe-seq used droplet microfluidic technology to enclose both the individual bacteria and the phage. It was not only found that *Bacteroidetes* common was the host species of phage crAssphage in this individual but also that acteroidetes common strain A was significantly associated with phage crAssphage more precisely at the strain level, which indicated the great potential of Microbe-seq in the study of host-phage.^83^ By virus labeling, microbial cells in human fecal samples were detected, host cells were labeled by viruses, host-phage pairs were isolated using fluorescence-activated cell sorting, and the whole genome was amplified and sequenced to identify 363 unique host-phage pairs, which expanded the known host-phage network of intestinal microbiota.^123^

Blattman et al examined 6663 *Staphylococcus aureus* single-cell transcriptomes and captured a rare subgroup of cells that received phage induction using prokaryotic PETRI-seq. This subgroup of *Staphylococcus aureus* is rich in the phage φSA3usa lysing gene, which is of great significance for clinical studies on phage induction and bacterial disease.^147^ The functional potential of bacteria and archaea has been widely studied in the human gut, but the functional potential of intestinal bacteriophages remains to be explored. It is now possible to make precise editing of gut microbes through the phage–host interaction mechanism. Studies have proved that phages can specifically target cytolytic *Escherichia faecalis* and the presence of cytolytic *Escherichia faecalis* is associated with the severity of liver disease and mortality in patients with alcoholic hepatitis, so phages play an important role in the treatment of alcoholic hepatitis.^148^ Meanwhile, phages are also associated with intestinal metabolome^149^ and tumor cells.^150^

## Revealing the role of microbes in the occurrence and treatment of diseases

### Pathogenic bacteria

Microbe-seq technology has been applied to the identification of pathogenic bacteria and susceptible cells and analysis of the potential pathogenesis. Ghaddar et al detected 19 bacteria associated with intestinal disease in pancreatic samples from two separate sets of large-scale pancreatic tube carcinoma single-cell RNA sequencing data (scPDA1 and scPDA2). The most abundant is *Campylobacter* spp (Campylobacter) which can cause intestinal or systemic inflammation. Tumor cells interact with bacteria more than any other cell types, which also suggests that bacteria are associated with tumor cell activity in the tumor microenvironment and that bacteria in tumors can induce immune cell infiltration and activation of the anti-tumor immune response at the single-cell level. What's more, the T-cell transcription profile in the pancreatic duct cancer microenvironment is very similar to that of infection, which partly explains the high level of inflammation in the pancreatic tumor microenvironment and the poor efficacy of immunotherapy.^151^

Microbe-seq detects scRNA sequences of both host cells and bacterial cells, which can analyze the differential host infection status caused by bacterial heterogeneity at the single-cell level. By ScdualNA-SEQ, Avital et al detected both host cells and *Salmonella*-derived cells at the single-cell level and identified class I and class II *Salmonella* with different transcriptional characteristics. After the two types of *Salmonella* infect host macrophages, three subgroups can be induced. Macrophages partially induced to be infected with *Salmonella* I, macrophages completely induced to be infected with *Salmonella* I, and macrophages completely induced to be infected with *Salmonella* II. The variation of *Salmonella* species was consistent with the linear progression of macrophages from a partially induced state to a fully induced state.^152^

### Probiotics

Probiotics generally promote health and fight disease. The effects of probiotics are usually directed towards the gut. In terms of promoting intestinal health, it mainly includes enhancing intestinal epithelial barrier function, increasing IgA levels in intestinal fluid, maintaining intestinal microbiome homeostasis, reducing intestinal pathogenic organisms by producing antibacterial components, and producing essential molecules.^153^ However, the efficacy of traditional probiotics is statistically insignificant, and the effects of probiotics vary from individual to individual.^154^^,^^155^ Microbe-seq is expected to provide molecular targets and research directions for specific probiotics as personalized medicine. Qiao et al found that a novel *Lactobacillus casei* strain BD5115 in yogurt secretes the metabolite 2-hydroxy-3-methylbutyrate, regulates the expression of MYC gene in mouse intestinal epithelial cells through MAFF/MBP1 interaction, and promotes the proliferation of intestinal epithelial cells using SCS.^156^ In this study, a new *Lactobacillus casei* BD5115 strain promoting intestinal epithelial cell proliferation was screened by SCS technology, and a new signaling pathway regulating MYC gene expression was also found. The molecular mechanism of probiotics and their probiotic metabolites regulating the state of host intestinal epithelial cells were described from the single-cell level.

### Clinical drug development related to microbial preparation

At present, in terms of drug research, SCS mainly focuses on high-throughput drug target screening, pharmacokinetic analysis, efficacy evaluation, drug resistance, and other studies at the single-cell level.^76^ Nowadays, SCS is the most abundant research in anti-tumor drugs. Taking the anti-tumor drug programmed cell death protein 1 (PD-1) inhibitor as an example, Krieg et al analyzed the immune cell subsets in the peripheral blood of patients with stage IV melanoma after 12 weeks of anti-PD-1 immunotherapy by high-dimensional single-cell mass spectrometry-based flow cytometry. It has been discovered that the frequency of CD14^+^ CD16^−^ HLA-DR^hi^ monocytes is correlated with the PD-1 immunotherapy response, and the number of monocytes in the immune cell population can successfully predict the therapeutic response effect of PD-1 inhibitors.^157^ Furthermore, in the research and development of new drug targets and drug resistance research, SCS allows us to trace the origin of tumor cells and compare the immediate response of tumor cells to different therapies, which greatly accelerates the research on tumor drug response and drug resistance, and makes us obtain a large number of disease-causing genes and medicinal genomic targets. Matthew T. Chang et al used TraCe-seq to trace the cells exposed to targeted therapy with different Epidermal growth factor receptor inhibitors that were responsive and resistant, and conducted differential gene analysis and time locus comparison as well. According to them, endoplasmic reticulum stress caused by inhibitor-bound epidermal growth factor receptors was crucial for achieving complete efficacy. This study suggested that a deeper understanding of the biochemical mechanisms by which inhibitors bind to epidermal growth factor receptors and endoplasmic reticulum stress induction could help the future development of small molecule drugs targeting epidermal growth factor receptors and other membrane-associated proteins.^158^ The development of SCS can not only enrich the target capacity from multiple perspectives but also narrow the target range more accurately.

Microbial biologics are based on the theory of microecologics as guidance, and they can regulate the balance of organism microecology using beneficial microorganisms or growth-promoting substances to the host. Microecological preparations play a good role in the treatment of diarrhea,^159^ irritable bowel syndrome, inflammatory bowel disease,^160^ and other diseases. Chemotherapeutic drugs and therapeutic genes often be delivered through polymer nano delivery systems, but currently, there are limitations, such as poor targeting ability and low concentration of drugs at the target location.^161^^,^^162^ However, bacteria have autonomy and high targeting ability, which can significantly improve the efficiency of drug delivery and drug concentration at the target site. Bacteria and their derivatives, such as bacterial outer membrane vesicles, spores, and bacterial ghosts, often act as vectors to participate in the drug delivery system and thus promote therapeutic effects.^163^, ^164^, ^165^ For example, bacterial outer membrane vesicles, which have neuroprotective effects and can cross the blood–brain barrier due to their antigenic properties, are a promising drug delivery vehicle for neurodegenerative diseases.^163^ Therapeutic phages have the same bactericidal effect as antibiotics in the treatment of bacterial infections, so they can solve the problem of antibiotic resistance with safety.^166^^,^^167^ However, the clinical application of microbiological agents is still in its infancy and many scientific problems remain to be solved. One of the most important things is to screen the appropriate target, such as whether therapeutic phages have targeted bacterial hosts and targeted bacterial gene action sites in the human body.^168^ Therefore, combined with the research direction of SCS in drug-related research, microbial SCS analysis is expected to provide disease targets for microbial biotherapy, such as therapeutic phages and probiotics.

## Conclusion

With the development of SCS and microbial sequencing, Microbe-seq came into being. SCS has been popularized in the field of microbial research for its ability to excavate unknown strains, explore intraspecies heterogeneity, and identify microbial functions at single-cell resolution. The current microbial SCS technologies and their limitations were summarized, which provided a valuable reference for the future application and optimization of microbial SCS. In addition, the diversity of detection objects gives microbial SCS great potential for application in the exploration of microbial-human host interactions and the role of microorganisms in disease progression. Our findings are expected to contribute to the promotion of microbial SCS and accelerate the optimization of the technology.

## Author contributions

Han Shuwen participated in the conception, design, and interpretation of the study; Wu Yinhang and Zhuang Jing drafted the manuscript; Song Yifei and Gao Xinyi designed and drew figures; Chu Jian reviewed and sorted out the literature; All authors read and approved the paper.

## Conflict of interests

The authors declare that no potential conflict of interests exists.

## Funding

This work was supported by the Key Research and Development Project of Zhejiang Province, China (No. 2022C03026), the Zhejiang Medical and Health Technology Project (China) (No. 2023RC274), and Public Welfare Technology Application Research Program of Huzhou, China (No. 2021GY15).
